# The effects of optic nerve sheath fenestration on visual function and OCT metrics in idiopathic intracranial hypertension: a retrospective study

**DOI:** 10.3389/fopht.2026.1752218

**Published:** 2026-03-26

**Authors:** Shadi Farabi Maleki, Milad Yousefi, Sepehr Fekrazad, Ataollah Asiayi, Reza Nabie, Maja Kostic

**Affiliations:** 1 Department of Ophthalmology, Nikookari Eye Hospital, Tabriz University of Medical Sciences, Tabriz, Iran; 2 Department of Mathematics and Computer Science, Tabriz University, Tabriz, Iran; 3 Department of Ophthalmology, Massachusetts Eye and Ear Infirmary, Harvard Medical School, Boston, MA, United States; 4 Department of Ophthalmology, Bascom Palmer Eye Institute, University of Miami, Miami, FL, United States

**Keywords:** idiopathic intracranial hypertension, optic nerve sheath fenestration, optical coherence tomography, retinal nerve fiber layer, ganglion cell layer

## Abstract

**Background:**

Idiopathic intracranial hypertension (IIH) is characterized by elevated intracranial pressure (ICP) without an identifiable cause and may lead to optic nerve damage and permanent vision loss. We evaluated longitudinal structural and functional outcomes after optic nerve sheath fenestration (ONSF) and how Optical Coherence Tomography (OCT)–visual function relationships evolve over early postoperative follow-up.

**Methods:**

This retrospective case series included 35 patients (70 eyes) with IIH who underwent bilateral ONSF. Structural and functional measures were assessed at baseline and at 6, 12, and 24 weeks postoperatively, including best-corrected visual acuity (BCVA) recorded on a 0–10 decimal chart, standard automated perimetry indices (mean deviation (MD), visual field index (VFI, pattern standard deviation), and spectral-domain OCT metrics (peripapillary retinal nerve fiber layer (RNFL) thickness and macular ganglion cell layer (GCL) measures. Pearson correlations were used to evaluate associations between OCT parameters and visual function at each time point.

**Results:**

OCT demonstrated significant reductions in optic nerve head swelling measures and peripapillary RNFL thickness over time (p < 0.05), consistent with postoperative improvement in papilledema. Macular GCL metrics also declined over follow-up, which may reflect delayed neuroaxonal loss and/or unmasking as edema resolves rather than edema resolution alone. BCVA and MD improved over follow-up, with several endpoints showing the largest change by approximately 12 weeks followed by stabilization through 24 weeks. Correlations between RNFL/macular metrics and visual field indices (VFI and MD) strengthened over time and were strongest by 24 weeks, consistent with increasing structure–function concordance as postoperative edema improves. Cup volume did not show statistically significant change.

**Conclusion:**

ONSF was associated with improvement in papilledema-related structural measures and visual function in vision-threatening IIH. OCT-derived parameters, particularly RNFL and macular ganglion cell metrics, are useful for longitudinal postoperative monitoring; early RNFL values may be confounded by edema, while later measurements better reflect axonal status and align more closely with visual function.

## 1 Introduction

Idiopathic intracranial hypertension (IIH) is a disorder of elevated intracranial pressure (ICP) without an identifiable cause that primarily affects young women and is strongly associated with female sex and obesity; proposed contributing mechanisms include metabolic and hormonal factors (1). Common clinical manifestations include headache, transient visual obscurations, and pulsatile tinnitus. However, papilledema remains the hallmark ocular finding and may ultimately lead to optic nerve atrophy and permanent vision loss, making preservation of visual function the central therapeutic goal in IIH management (2, 3).

Despite advances in diagnosis and treatment, IIH remains challenging to monitor. Visual deterioration may occur insidiously and currently used assessment methods have limitations. Repeated lumbar punctures are invasive and impractical for longitudinal follow-up, and clinical grading systems such as the Frisén scale are subject to interobserver variability (4). These limitations underscore the need for objective, quantitative biomarkers capable of reliably tracking optic nerve status over time.

Optical coherence tomography (OCT) provides non-invasive, high-resolution structural assessment of the optic nerve head and retina. It enables direct quantification of peripapillary retinal nerve fiber layer (RNFL) thickness, as well as macular ganglion cell layer (GCL) and ganglion cell complex (GCC) metrics that reflect neuroaxonal integrity (2). Additional retinal features, including retinal pigment epithelium/Bruch’s membrane (RPE/BM) configuration and optic nerve head morphology, can also be evaluated using OCT (5). These parameters allow clinicians to detect subtle structural changes, monitor treatment response, and potentially anticipate functional outcomes. However, OCT interpretation in IIH is not without limitations. In the setting of severe papilledema, segmentation errors may occur, and variability in scan acquisition protocols and normative databases can affect measurement precision (6, 7). Importantly, these limitations are most pronounced during marked swelling. Once edema improves, OCT measurements are generally highly repeatable and well suited for longitudinal monitoring of structural change.

A critical clinical challenge lies in distinguishing resolving papilledema from evolving neuroaxonal loss. Early RNFL thickening due to axoplasmic stasis may mask underlying axonal injury, whereas subsequent RNFL thinning during recovery may represent a combination of edema resolution and delayed or unmasked atrophy. Macular GCL metrics are particularly valuable in this context, as they may better reflect neuronal integrity during later stages of recovery (6, 7). Understanding this dynamic is essential when interpreting postoperative OCT changes.

Consensus guidance emphasizes vision preservation, with optic nerve sheath fenestration (ONSF) generally reserved for vision-threatening IIH that progresses despite optimized medical therapy (8, 9). Clinically, ONSF is considered when visual function worsens in the setting of moderate-to-severe papilledema or fulminant presentations, while systemic contributors such as weight and ICP drivers are addressed in parallel (10).

Our study evaluates longitudinal OCT-derived structural changes following ONSF and correlates them with visual outcomes over time. While prior studies have examined structural and functional outcomes after ONSF, detailed characterization of early postoperative structure–function coupling remains incompletely defined, particularly across serial time points during edema resolution.

## 2 Materials and methods

This study was a retrospective, observational, non-comparative case series conducted at Nikookari Eye Center, Tabriz University of Medical Sciences, between January 2020 and December 2022. All patients provided written informed consent for use of their anonymized clinical data. The study adhered to the tenets of the Declaration of Helsinki. The study was approved by the Ethics Committee of Tabriz University of Medical Sciences (ethics code IR.TBZMED.REC.1402.336). Inclusion criteria required a diagnosis of IIH documented in the medical record using the modified Dandy criteria (11), (symptoms/signs of raised ICP, normal neuroimaging and Cerebrospinal fluid (CSF) composition, and elevated lumbar puncture opening pressure ≥ 250 mm H_2_O). We used these criteria because they were the diagnostic framework consistently documented during the study period; contemporary consensus criteria [e.g., Mollan 2018 (10)] emphasize similar core elements (exclusion of secondary causes and confirmed elevated opening pressure). All patients exhibited progressive visual deterioration and persistent papilledema despite maximal medical therapy. Exclusion criteria included ocular comorbidities affecting OCT or VF (e.g., glaucoma, diabetic retinopathy, high myopia), prior CSF shunting, incomplete follow-up, or lack of consent.

For all eligible patients, clinical data were retrospectively extracted from the electronic medical record, and OCT metrics were obtained from the Topcon imaging database at baseline and at 6, 12, and 24 weeks postoperatively. Standard automated perimetry was performed using the Humphrey Field Analyzer (Carl Zeiss Meditec, Jena, Germany) with the 24–2 program and SITA Standard strategy; visual field indices included mean deviation (MD, dB), visual field index (VFI, %), and pattern standard deviation (PSD, dB). Spectral-domain OCT imaging was acquired using the Topcon DRI OCT Triton (Topcon Corporation, Tokyo, Japan) (12). All scans were reviewed for segmentation accuracy, with manual correction performed, when necessary, by a trained grader masked to clinical time points. Macular segmentation outputs were recorded as reported by the device. Ganglion Cell Layer plus (GCL+) was defined as the combined thickness from the inner limiting membrane (ILM) to the outer boundary of the inner plexiform layer (IPL), encompassing the RNFL, ganglion cell layer (GCL), and inner plexiform layer. Ganglion Cell Complex (GCL++) was defined as the combined thickness from the ILM to the outer boundary of the inner nuclear layer (INL), incorporating the RNFL, GCL, IPL, and INL. Measurements were reported as average (total), superior, and inferior macular sectors.

All patients underwent bilateral optic nerve sheath fenestration using a standardized medial transconjunctival approach performed by a single experienced surgeon (R.N.) to ensure procedural consistency. The optic nerve sheath was accessed via blunt dissection through the medial rectus muscle, followed by creation of a 3–5 mm fenestration for cerebrospinal fluid decompression.

Baseline evaluations, including best-corrected visual acuity (BCVA; recorded on a 0–10 decimal acuity chart where 10 = 20/20 equivalent), papilledema grading, OCT imaging, and visual field testing, were repeated at postoperative visits at 6, 12, and 24 weeks.

Statistical analyses were conducted in Python 3.10 using parametric or nonparametric tests as appropriate. For longitudinal comparisons to baseline at each postoperative time point, normality was assessed visually using Q–Q plots and by Shapiro–Wilk testing. We then used paired tests (paired t-test for approximately normally distributed measures; Wilcoxon signed-rank otherwise). Analyses were performed at the eye level. Because both eyes from the same patient were included, observations are not fully independent; inferential p-values should therefore be interpreted cautiously. Correlation analyses were considered exploratory and intended to describe longitudinal structure–function coupling.

## 3 Results

### 3.1 Demographics and baseline characteristics

Thirty-five patients (70 eyes; mean age 33.3 ± 11.7 years; 91.5% female) were analyzed. The mean follow-up duration was 16.8 months (range: 6–46 months). All included patients had data available at baseline and the scheduled postoperative visits for the primary measures. Also, all eligible eyes, including those with postoperative complications, were retained in the longitudinal analyses (**Table 1**).

**Table 1:** Baseline demographic and clinical characteristics.

Characteristic	Mean ± SD/n (%)	Range
Age (years)	33.3 ± 11.7	17 – 69
Sex		
Female	32 (91.5%)	–
Male	3 (8.5%)	–
Follow-up duration (months)	16.8	6 – 46
Number of eyes	70	–
Data completeness	No missing data or exclusions	–

### 3.2 Visual function and OCT parameters

#### 3.2.1 RNFL parameters

There was a significant reduction in total RNFL thickness from 173.9 ± 104.5 µm at baseline to 121.7 ± 70.2 µm at 6 weeks (p < 0.05), 100.4 ± 32.2 µm at 12 weeks (p < 0.05), and 97.6 ± 23.8 µm at 24 weeks (p < 0.001). Superior RNFL thickness decreased from 194.7 ± 139.5 µm to 135.5 ± 82.4 µm at 6 weeks (p < 0.05), 112.7 ± 42.7 µm at 12 weeks (p < 0.05), and 107.4 ± 33.8 µm at 24 weeks (p < 0.05). Inferior RNFL thickness also showed a significant reduction from 232.3 ± 116.0 µm preoperatively to 166.7 ± 74.4 µm at 6 weeks (p < 0.05), 145.1 ± 50.4 µm at 12 weeks (p < 0.001), and 138.7 ± 37.5 µm at 24 weeks (p < 0.001).

The rim area significantly decreased from 4.28 ± 2.18 mm² to 2.68 ± 1.04 mm² at 6 weeks, 2.46 ± 0.74 mm² at 12 weeks, and 2.30 ± 0.57 mm² at 24 weeks (p < 0.001 for all). Disc area also significantly declined from 4.66 ± 2.79 mm² to 2.78 ± 0.98 mm² at 6 weeks, 2.54 ± 0.70 mm² at 12 weeks, and 2.43 ± 0.50 mm² at 24 weeks (p < 0.001). Linear and vertical cup-to-disc ratios showed a modest but statistically significant increase by 24 weeks (p < 0.05), while cup volume remained unchanged (p ≥ 0.05).

GCL++ thickness showed a significant reduction from 104.8 ± 17.1 µm preoperatively to 100.7 ± 17.0 µm at 6 weeks (p < 0.05), 97.0 ± 15.2 µm at 12 weeks, and 96.0 ± 13.7 µm at 24 weeks (p < 0.001). GCL+ thickness also significantly decreased over time, from 66.3 ± 12.0 µm to 65.7 ± 12.3 µm at 6 weeks, 63.5 ± 12.2 µm at 12 weeks, and 63.3 ± 9.7 µm at 24 weeks (p < 0.05 at all-time points). Macular volume significantly decreased from 7.81 ± 0.62 mm³ at baseline to 7.56 ± 0.58 mm³ at 6 weeks (p < 0.001), 7.54 ± 0.60 mm³ at 12 weeks (p < 0.001), and 7.61 ± 0.47 mm³ at 24 weeks (p < 0.05). Central macular thickness showed no statistically significant changes throughout the follow-up period (p ≥ 0.05).

#### 3.2.2 Visual field and function parameters

MD improved significantly from –12.6 ± 10.9 dB at baseline to –10.0 ± 11.3 dB at 6 weeks (p < 0.001) and –10.1 ± 9.4 dB at 12 weeks (p < 0.05), but the change at 24 weeks (–9.3 ± 9.0 dB) was not statistically significant (p ≥ 0.05). Visual field index (VFI) increased from 72.4 ± 31.2% to 81.4 ± 22.6% at 24 weeks, but this change was not statistically significant (p ≥ 0.05). PSD remained unchanged across all time points (p ≥ 0.05). The attenuation of MD improvement by 24 weeks may reflect stabilization after early postoperative recovery and/or expected test–retest variability in automated perimetry.

#### 3.2.3 Visual acuity

Significant improvement in VA was observed, increasing from 8.41 ± 2.59 pre-operatively to 9.32 ± 1.72 at 12 weeks (p < 0.05) and 9.35 ± 1.71 at 24 weeks. VA improved modestly over follow-up, with most change observed by 12 weeks and stability thereafter.

### 3.3 Papilledema grading (Frisén Score)

Frisén score showed a significant and consistent reduction from 3.91 ± 1.18 at baseline to 1.44 ± 1.44 at 6 weeks, 0.30 ± 0.61 at 12 weeks, and 0.03 ± 0.17 at 24 weeks (p < 0.0001 for all).

**Figure 1:**
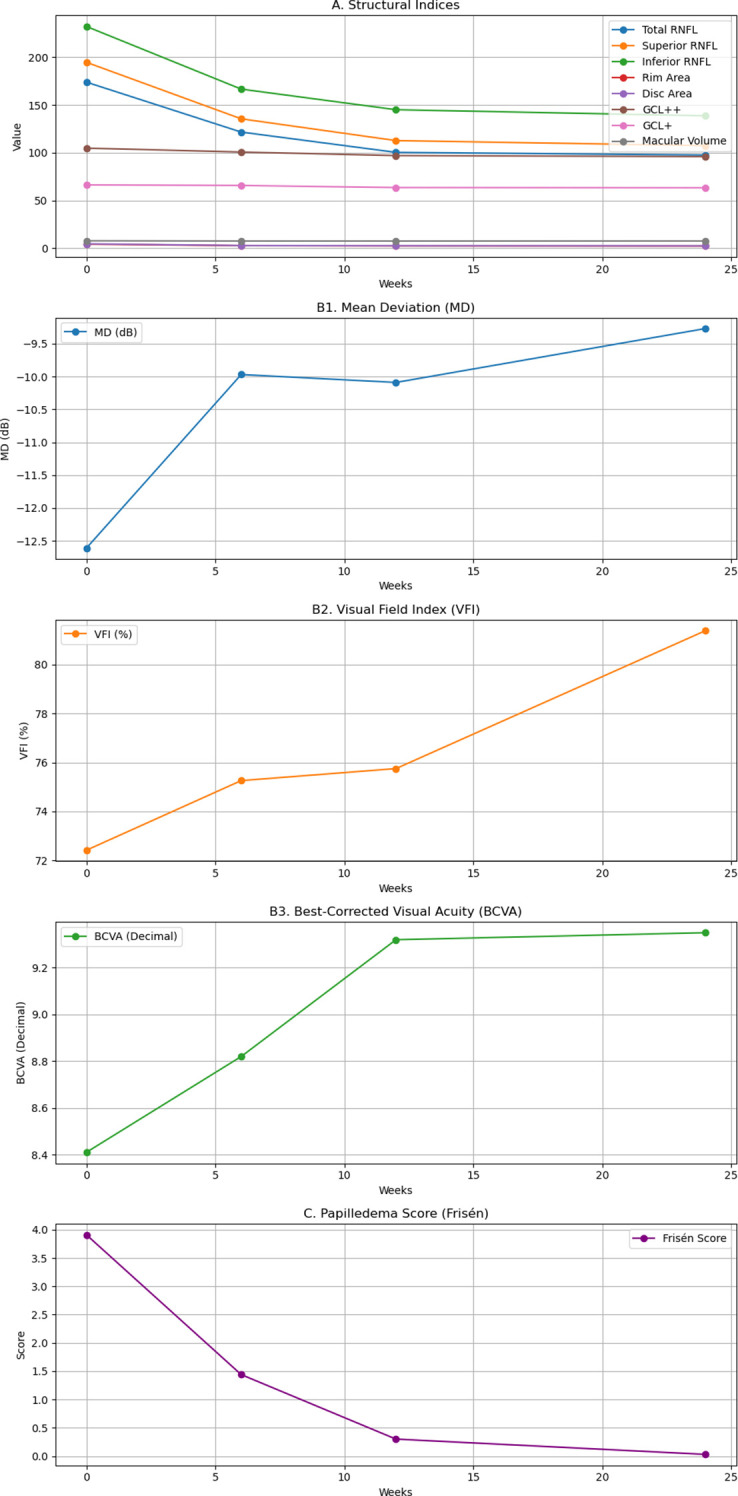
Longitudinal changes in **(A)** structural indices, **(B1)** mean deviation (MD), **(B2)** visual field index (VFI), **(B3)** best-corrected visual acuity (BCVA, Decimal), and **(C)** papilledema score (Frisén) at baseline and follow-up visits. RNFL, retinal nerve fiber layer; GCL, ganglion cell layer.

Across multiple structural endpoints, the largest absolute change occurred by 12 weeks with smaller incremental changes thereafter. For example, total RNFL decreased from 173.9 µm at baseline to 100.4 µm at 12 weeks and 97.6 µm at 24 weeks, and Frisén grade decreased from 3.91 at baseline to 0.30 at 12 weeks and 0.03 at 24 weeks. However, not all measures changed monotonically over time; for example, macular average thickness and inferior GCL++ showed small fluctuations between 12 and 24 weeks, and MD demonstrated modest interval variability, consistent with expected measurement variability and/or evolving edema–atrophy balance. (**Table 2**; **Figure 1**).

**Table 2:** Evaluation of visual function and OCT parameters before and after surgery.

Parameters	Pre-operation (mean ± SD)	6-weeks (mean ± SD)	P value	12-weeks (mean ± SD)	P value	24-weeks (mean ± SD)	P value
RNFL Total (µm)	173.94 ± 104.47	121.66 ± 70.20	p < 0.05	100.41 ± 32.21	p < 0.05	97.59 ± 23.81	p < 0.001
RNFL Superior (µm)	194.74 ± 139.54	135.54 ± 82.41	p < 0.05	112.74 ± 42.71	p < 0.05	107.41 ± 33.81	p < 0.05
RNFL Inferior (µm)	232.29 ± 116.05	166.66 ± 74.43	p < 0.05	145.09 ± 50.40	p < 0.001	138.67 ± 37.50	p < 0.001
Rim Area (mm²)	4.28 ± 2.18	2.68 ± 1.04	p < 0.001	2.46 ± 0.74	p < 0.001	2.30 ± 0.57	p < 0.001
Disc Area (mm²)	4.66 ± 2.79	2.78 ± 0.98	p < 0.001	2.54 ± 0.70	p < 0.001	2.43 ± 0.50	p < 0.001
C/D Linear	0.06 ± 0.16	0.12 ± 0.17	p < 0.05	0.11 ± 0.15	p < 0.05	0.14 ± 0.17	p < 0.05
C/D Vertical	0.06 ± 0.16	0.13 ± 0.17	p < 0.05	0.11 ± 0.15	p < 0.05	0.14 ± 0.17	p < 0.05
Cup Volume	0.01 ± 0.02	0.01 ± 0.02	p ≥ 0.05	0.01 ± 0.02	p ≥ 0.05	0.01 ± 0.03	p ≥ 0.05
Macular GCL++ Total (µm)	104.84 ± 17.13	100.74 ± 16.95	p < 0.05	97.00 ± 15.24	p < 0.001	96.00 ± 13.69	p < 0.001
Macular GCL++ Superior (µm)	103.42 ± 18.57	99.43 ± 18.55	p < 0.05	95.24 ± 16.40	p < 0.001	94.38 ± 14.83	p < 0.001
Macular GCL++ Inferior (µm)	106.35 ± 17.15	102.17 ± 16.75	p < 0.05	99.21 ± 15.44	p < 0.001	99.81 ± 17.83	p < 0.05
Macular GCL+ Total (µm)	66.32 ± 12.02	65.74 ± 12.27	p < 0.05	63.52 ± 12.19	p < 0.05	63.31 ± 9.66	p < 0.05
Macular GCL+ Superior(µm)	65.10 ± 12.90	64.60 ± 13.41	p < 0.05	62.70 ± 13.12	p < 0.05	62.46 ± 10.29	p < 0.05
Macular GCL+ Inferior (µm)	67.65 ± 12.09	67.09 ± 12.15	p < 0.05	64.73 ± 11.80	p < 0.05	64.31 ± 9.71	p < 0.05
Macular Average	276.82 ± 22.41	267.85 ± 20.56	p < 0.001	258.97 ± 25.23	p ≥ 0.05	269.33 ± 16.81	p < 0.05
Macular Central	189.91 ± 39.20	177.12 ± 18.39	p ≥ 0.05	183.27 ± 16.36	p ≥ 0.05	182.83 ± 13.17	p ≥ 0.05
Macular Volume	7.81 ± 0.62	7.56 ± 0.58	p < 0.001	7.54 ± 0.60	p < 0.001	7.61 ± 0.47	p < 0.05
MD (dB)	-12.61 ± 10.93	-9.97 ± 11.28	p < 0.001	-10.09 ± 9.35	p < 0.05	-9.27 ± 8.97	p ≥ 0.05
PSD (dB)	7.16 ± 4.15	6.24 ± 3.87	p ≥ 0.05	7.75 ± 4.08	p ≥ 0.05	6.22 ± 3.31	p ≥ 0.05
VFI *(%)*	72.42 ± 31.21	75.26 ± 31.50	p ≥= 0.05	75.75 ± 27.77	p ≥ 0.05	81.38 ± 22.58	p ≥ 0.05
VA	8.41 ± 2.59	8.82 ± 2.22	p < 0.05	9.32 ± 1.72	p < 0.05	9.35 ± 1.71	p < 0.05
Frisén *(grade)*	3.91 ± 1.18	1.44 ± 1.44	p < 0.0001	0.30 ± 0.61	p < 0.0001	0.03 ± 0.17	p < 0.0001

### 3.4 Correlations between visual and OCT parameters

#### 3.4.1 RNFL total

A strong positive correlation was observed between RNFL total thickness and rim area preoperatively (R = 0.8815, p < 0.0001), which remained statistically significant at 6-, 12-, and 24-weeks following surgery, although with decreasing strength over time. The correlation between RNFL total thickness and VFI was not statistically significant at baseline but became significant by 12 weeks (R = 0.4127, p < 0.05) and showed a marked increase by 24 weeks (R = 0.7732, p < 0.0001). Similarly, RNFL total thickness demonstrated a moderate correlation with macular volume at baseline (R = 0.3443, p < 0.05), which increased at each follow-up visit, reaching a peak at 24 weeks (R = 0.5763, p < 0.001).

Strong correlations were also found between RNFL total thickness and macular ganglion cell layer metrics. At 24 weeks, RNFL total thickness was significantly correlated with GCL+ thickness (R = 0.6785, p < 0.0001) and more strongly with GCL++ thickness (R = 0.7916, p < 0.0001). These findings were consistent across superior and inferior macular sectors.

No statistically significant correlations were found between RNFL total thickness and cup volume at any time point (p ≥ 0.05). The correlation between RNFL total thickness and Frisén grade was moderate preoperatively (R = 0.3974, p < 0.05) but progressively decreased following surgery. There was no significant correlation between RNFL total thickness and VA at any time point (p ≥ 0.05). MD did not correlate significantly with RNFL thickness at baseline but became significantly associated at 12 weeks and was strongly correlated by 24 weeks (R = 0.6317, p < 0.0001) (**Table 3**).

**Table 3:** Correlation between visual and OCT parameters.

Parameters	Pre-operation		6-Weeks after surgery		12-Weeks after surgery		24-weeks after surgery	
	*Pearson correlation (R)*	*P-value*	*Pearson correlation (R)*	*P-value*	*Pearson correlation (R)*	*P-value*	*Pearson correlation (R)*	*P-value*
RNFL Total & Rim Area	0.8815	p < 0.0001	0.6921	p < 0.0001	0.7544	p < 0.0001	0.7005	p < 0.0001
RNFL Total & VFI	-0.0104	p ≥ 0.05	0.2748	p ≥ 0.05	0.4127	p < 0.05	0.7732	p < 0.0001
RNFL Total & Macular Volume	0.3443	p < 0.05	0.4105	p < 0.05	0.5132	p < 0.05	0.5763	p < 0.001
RNFL Total & Macular GCL+	0.3558	p < 0.05	0.4344	p < 0.05	0.5287	p < 0.001	0.7118	p < 0.0001
RNFL Total & Macular GCL++	0.5494	p < 0.001	0.4885	p < 0.05	0.6225	p < 0.0001	0.7916	p < 0.0001
RNFL Total & Cup Volume	-0.0718	p ≥ 0.05	-0.0950	p ≥ 0.05	0.0046	p ≥ 0.05	0.0197	p ≥ 0.05
RNFL Total & Frisén	0.3974	p < 0.05	0.3465	p < 0.05	0.0585	p ≥ 0.05	0.2854	p ≥ 0.05
RNFL Total & VA	0.0028	p ≥ 0.05	0.2107	p ≥ 0.05	0.2924	p ≥ 0.05	0.1017	p ≥ 0.05
RNFL Total & MD	-0.2171	p ≥ 0.05	0.0331	p ≥ 0.05	0.3980	p < 0.05	0.6317	p < 0.0001
Rim Area & VFI	0.0086	p ≥ 0.05	0.4420	p < 0.05	0.3623	p < 0.05	0.5637	p < 0.001
Rim Area & Cup Volume	-0.0428	p ≥ 0.05	-0.3545	p ≥ 0.05	-0.2632	p ≥ 0.05	-0.4464	p ≥ 0.05
Rim Area & Macular Volume	0.3791	p < 0.05	0.3518	p < 0.05	0.3804	p < 0.05	0.2001	p ≥ 0.05
Rim Area & Macular GCL+	0.2579	p ≥ 0.05	0.3212	p ≥ 0.05	0.4071	p < 0.05	0.3550	p < 0.05
Rim Area & Macular GCL++	0.4467	p < 0.05	0.3506	p < 0.05	0.4622	p < 0.05	0.4275	p < 0.05
Rim Area & Frisén	0.3828	p < 0.05	0.2912	p ≥ 0.05	0.2596	p ≥ 0.05	0.1437	p ≥ 0.05
Rim Area & VA	0.0043	p ≥ 0.05	0.1782	p ≥ 0.05	0.0855	p ≥ 0.05	-0.0708	p ≥ 0.05
Rim Area & MD	-0.1764	p ≥ 0.05	0.3341	p ≥ 0.05	0.3516	p ≥ 0.05	0.3040	p ≥ 0.05
Macular Volume & Cup Volume	0.0077	p ≥ 0.05	0.1698	p ≥ 0.05	0.1865	p ≥ 0.05	0.3185	p ≥ 0.05
Macular Volume & VFI	0.5180	p < 0.05	0.6352	p < 0.0001	0.7503	p < 0.0001	0.7131	p < 0.0001
Macular Volume & Frisén	0.0445	p ≥ 0.05	0.2392	p ≥ 0.05	0.1151	p ≥ 0.05	0.1126	p ≥ 0.05
Macular Volume & VA	0.3638	p < 0.05	0.4397	p < 0.05	0.5293	p < 0.001	0.2381	p ≥ 0.05
Macular Volume & MD	0.4250	p < 0.05	0.5373	p < 0.001	0.7357	p < 0.0001	0.6013	p < 0.001
Macular GCL+ & Cup Volume	-0.4406	p < 0.05	0.1659	p ≥ 0.05	0.2122	p ≥ 0.05	0.2971	p ≥ 0.05
Macular GCL+ & VFI	0.4004	p < 0.05	0.6671	p < 0.0001	0.7005	p < 0.0001	0.7637	p < 0.0001
Macular GCL+ & Frisén	0.2442	p ≥ 0.05	0.1641	p ≥ 0.05	0.0367	p ≥ 0.05	0.0276	p ≥ 0.05
Macular GCL+ & VA	0.3336	P < 0.05	0.5396	P < 0.05	0.4775	P < 0.05	0.2301	P ≥ 0.05
Macular GCL+ & MD	0.2998	p ≥ 0.05	0.5821	p < 0.001	0.6880	p < 0.0001	0.5702	p < 0.001
Macular GCL++ & Cup Volume	-0.4368	p < 0.05	0.1280	p ≥ 0.05	0.1864	p ≥ 0.05	0.2561	p ≥ 0.05
Macular GCL++ & VFI	0.2812	p ≥ 0.05	0.5897	p < 0.001	0.6760	p < 0.0001	0.8279	p < 0.0001
Macular GCL++ & Frisén	0.3145	p ≥ 0.05	0.2621	p ≥ 0.05	0.0929	p ≥ 0.05	0.0946	p ≥ 0.05
Macular GCL++ & VA	0.1192	p ≥ 0.05	0.3936	p < 0.05	0.4164	p < 0.05	0.2173	p ≥ 0.05
Macular GCL++ & MD	0.2015	p ≥ 0.05	0.4922	p < 0.05	0.6629	p < 0.0001	0.6251	p < 0.0001
Cup Volume & Frisén	0.2094	p ≥ 0.05	0.0218	p ≥ 0.05	0.0428	p ≥ 0.05	-0.1011	p ≥ 0.05
Cup Volume & VFI	0.0237	p ≥ 0.05	-0.1570	p ≥ 0.05	-0.0953	p ≥ 0.05	0.0580	p ≥ 0.05
Cup Volume & VA	0.1675	p ≥ 0.05	0.1444	p ≥ 0.05	0.1523	p ≥ 0.05	0.0956	p ≥ 0.05
Cup Volume & MD	0.0083	p ≥ 0.05	-0.1296	p ≥ 0.05	-0.0899	p ≥ 0.05	0.1494	p ≥ 0.05
VA & Frisén	0.0864	p ≥ 0.05	-0.2445	p ≥ 0.05	-0.3425	p < 0.05	0.0519	p ≥ 0.05
VA & VFI	0.5544	p < 0.001	0.4820	p < 0.05	0.4393	p < 0.05	0.0958	p ≥ 0.05

#### 3.4.2 Rim area

Rim area demonstrated no statistically significant correlation with VFI preoperatively. However, by 6 weeks postoperatively, a significant correlation emerged (R = 0.4420, p < 0.05) and strengthened further by 24 weeks (R = 0.5637, p < 0.001). Correlations between rim area and macular volume were consistently positive throughout the study period, with varying degrees of significance. At 12 weeks, a moderate correlation was observed (R = 0.4902, p < 0.001). No significant correlation was found between rim area and VA at any time point (p ≥ 0.05) (**Table 3**).

#### 3.4.3 Macular volume

Macular volume showed a significant correlation with VFI as early as baseline, which increased progressively through the postoperative period. The strongest correlation was observed at 12 weeks (R = 0.7503, p < 0.0001), and this association remained significant at 24 weeks. Initial correlations between macular volume and VA were statistically significant (R = 0.4645, p < 0.01), but this association weakened and became non-significant by 24 weeks. A strong correlation was also found between macular volume and MD at 12 weeks (R = 0.7357, p < 0.001), which persisted through the 24-week follow-up (**Table 3**).

#### 3.4.4 Macular GCL+ and GCL++

Macular GCL+ thickness demonstrated a significant correlation with VFI beginning at 6 weeks (R = 0.6671, p < 0.0001), which increased further by 24 weeks (R = 0.7637, p < 0.0001). GCL++ thickness showed a similar trend, with a strong and statistically significant correlation with VFI emerging by 12 weeks and peaking at 24 weeks (R = 0.8279, p < 0.0001). These correlations were consistent across superior and inferior regions (**Table 3**).

#### 3.4.5 Cup volume correlations

Cup volume did not exhibit any statistically significant correlation with functional parameters such as VFI, MD, or VA, nor with structural parameters including RNFL thickness, rim area, or macular metrics at any postoperative time point (p ≥ 0.05).

Overall, OCT–visual field correlations strengthened progressively across follow-up, consistent with increasing structure–function concordance as postoperative edema improves, and OCT thickness metrics better reflect axonal status (**Table 3**).

### 3.5 Clinical complications and representative cases

One patient, who had previously undergone surgery for a hypophyseal adenoma, presented with recurrent optic disc swelling 14 months postoperatively. Delayed follow-up and missed appointments contributed to a lack of timely intervention. Two other patients required ventriculoperitoneal shunt placement due to sustained elevation in ICP, despite undergoing ONSF.

One patient developed optic disc atrophy six months after surgery, and another exhibited intraretinal hemorrhages and choroidal folds that ultimately led to optic atrophy. A separate patient experienced increased ICP five months post-ONSF and subsequently required a shunt procedure. In another case, irregular OCT assessments resulted in delayed detection of optic nerve changes, and the patient later developed sixth nerve palsy and esotropia. Lastly, a patient with a seven-year history of chronic IIH showed significant optic nerve atrophy one year following ONSF.

## 4 Discussion

Our findings support the efficacy of ONSF in improving papilledema-related optic nerve head swelling measures and stabilizing or improving visual function in vision-threatening IIH. Mechanistically, ONSF is thought to locally reduce the trans-laminar pressure gradient at the optic nerve head, which may protect the optic nerve even when systemic ICP drivers require ongoing management; our strengthening structure–function correlations by 12–24 weeks are consistent with this rationale (10).

El-Masri et al. (13) conducted a study including patients who underwent ONSF, cerebral venous sinus thrombosis (CVST), or other conditions. Following ONSF, 84% of patients showed improvement or stabilization in BCVA, and 50% achieved a VA of 6/6 or better. RNFL thickness, VF, and optic disc grade generally improved, especially up to day 360. These findings align with our study results. Both studies highlight that prior medical or surgical treatments for IIH may complicate interpretation of peripapillary RNFL outcomes, as treatment-related edema resolution, pre-existing axonal loss, or prior surgical alteration of optic nerve head morphology may influence postoperative RNFL thickness independently of current disease activity.

Several interventional approaches are available for refractory or vision-threatening IIH, each with distinct mechanisms and risk profiles. CSF diversion procedures, including lumbar puncture (LP) and ventriculoperitoneal (VP) shunting, can lower ICP and often improve headache but carry risks of device failure, infection, over-drainage, and revision. Venous sinus stenting targets a documented trans-stenotic pressure gradient and may improve papilledema and headache in selected patients but requires careful selection and carries endovascular risks. By contrast, ONSF is primarily a vision-protective, eye-directed procedure that targets papilledema and may stabilize or improve visual function; limitations include variable effects on headache and the fact that postoperative OCT improvement does not necessarily indicate global ICP normalization. Procedure-specific risks include diplopia/extraocular motility disturbance, orbital hemorrhage, and incomplete or recurrent papilledema requiring additional intervention. Ultimately, the choice of procedure should reflect phenotype (vision-threatening vs headache-dominant), venous physiology, and comorbidity (14, 15).

Similarly, Ling Dai et al. (16) conducted a retrospective evaluation of VF, MD, RNFL, papilledema grade, and VA before and after ONSF in IIH patients. They reported a steady improvement in MD and VF over 12 months, with RNFL thickness decreasing significantly in both operated and non-operated eyes. Although fewer OCT parameters were analyzed in their study, our results are in agreement regarding RNFL and MD changes. Our study further explored inter-parameter correlations and their relevance to visual function.

A significant and sustained reduction in RNFL thickness was observed following ONSF, with mean total RNFL measurements decreasing steadily from baseline through 24 weeks. This trend reflects a progressive resolution of disc edema, consistent with local decompression after fenestration and postoperative improvement in papilledema. Both superior and inferior RNFL segments followed this pattern, though the inferior segment demonstrated a more pronounced and rapid response. These findings suggest regional differences in susceptibility to pressure-induced swelling and responsiveness to surgical decompression. Inferior structural vulnerability can be clinically relevant to superior field loss; however, we did not perform pointwise or cluster-based perimetry analyses, so topographic structure–function mapping should be evaluated in future studies.

Kaufhold et al. (17) demonstrated that ONH volume is markedly elevated in IIH patients compared to controls even with similar RNFL thickness, suggesting ONH volume as a sensitive diagnostic and treatment response metric. Their findings that ONH swelling occurs more superficially than in height support our data, which showed significant postoperative ONH volume reduction, reinforcing its utility in tracking IIH progression and response to intervention. Our results align with the study by Huang-Link et al. (18), which showed that IIH patients with papilledema had elevated RNFL thickness and CSF pressure, both of which decreased following CSF aspiration. In contrast, patients without papilledema had normal RNFL values with no significant change post-aspiration. Their findings suggest that rim area, rim thickness, and decreased cup volume are sensitive markers for disease activity and recurrence. Our study similarly supports RNFL and rim thickness as effective metrics for monitoring IIH, including in the absence of overt papilledema.

We observed a significant decline in macular GCL++ and GCL+ thickness in the superior, inferior, and total sectors across all time points, which parallels the results of Malm Hagen et al. (19). Their prospective study found an inverse correlation between opening pressure on lumbar puncture and baseline ganglion cell loss, indicating that elevated opening pressure is associated with greater retinal GC degeneration. Importantly, the progressive decline in macular GCL+ and GCL++ thickness over follow-up is unlikely to represent edema resolution alone and more plausibly reflects delayed neuroaxonal loss and/or unmasking of ganglion cell loss as papilledema resolves. This has prognostic implications and supports careful interpretation of macular metrics as indicators of neuronal integrity during recovery. In contrast, Dreesbach et al. (20), reported no difference in macular GCL thickness between IIH patients and controls, suggesting an acute, rather than chronic, disease state. This discrepancy may reflect differences in disease chronicity: macular GCL thinning is more consistently observed in chronic or recurrent disease and may be less apparent in acute presentations or cohorts with shorter duration Also, their study lacked longitudinal follow-up and did not include postoperative evaluation. Additionally, their finding of significant correlation between GCL and both BCVA and MD supports the clinical value of macular GCL metrics, even in atrophic regions.

No significant changes in PSD were observed in our cohort throughout the 24-week follow-up, which is consistent with findings by Hagen et al. (19) showing MD improvement only after six months, particularly in patients who received earlier surgical intervention. In our study, MD improved primarily during the early postoperative period, with no significant changes beyond 12 weeks. This trend is consistent with findings from Landau et al. (21), who reported improved VFs and MD in both operated and non-operated pediatric ONSF groups. Notably, the operated group demonstrated greater RNFL improvement, suggesting that while ICP-lowering interventions generally support visual function recovery, ONSF may facilitate a more rapid postoperative improvement, particularly in the immediate recovery phase.

We observed progressive strengthening of correlations between OCT thickness metrics (RNFL and macular ganglion cell measures) and visual field indices (MD, VFI) over follow-up. This pattern is consistent with increasing structure–function concordance as postoperative edema resolves, such that OCT measures may better reflect underlying axonal status at later time points. These findings should be interpreted as associative rather than causal, particularly given the retrospective design and multiple comparisons (17). Moreover, early postoperative OCT changes must be interpreted in the context of papilledema resolution, where apparent structural improvement may partially reflect edema normalization and unmasking of pre-existing axonal loss rather than pure neuroaxonal recovery. The strong correlation between macular GCL thickness and VFI therefore reflects evolving structure–function alignment rather than definitive mechanistic recovery. Ganglion cells are integral to the transmission of visual signals, and their health directly affects visual function (22). As ICP decreases, it’s possible that axoplasmic flow will return and cellular stress will decrease, potentially allowing for stabilization of ganglion cell stress and improved functional performance over time (23). Additionally, the slow strengthening of connections over time points to a functional recovery that is slower than structural changes. This delay may reflect the time required for functional recovery, reduced axoplasmic stasis, and neuroadaptive processes within the visual pathway following structural stabilization. The complex cerebral circuitry involved in visual processing may take longer to stabilize and react to these advancements, whereas the optic nerve and ganglion cell structures may recover from structural stress rather quickly (24). Conversely, the lack of significant correlations between certain OCT parameters, such as cup volume and visual function, highlights the selective relevance of specific structural markers in monitoring disease status. Our results highlight the complex relationship between visual function and structural change in the optic nerve, suggesting that surgical management of IIH relieves pressure while allowing gradual functional stabilization over time. In order to completely comprehend the dynamics of recovery and improve patient outcomes, it is imperative that both structural and functional characteristics be continuously monitored during the post-operative phase.

This study has several limitations. Its retrospective design and lack of a control group limit causal inference. Both eyes were analyzed independently, which may introduce inter-eye correlation bias. OCT segmentation quality was not formally graded, although manual correction was performed when necessary. Additionally, independent assessment for Frisén grading interpretation was not conducted, reducing interpretive reliability. Also, VF testing reliability, while monitored, may still vary in patients with reduced vision or fixation instability. Furthermore, while the 24-week follow-up captures early-to-midterm outcomes, longer-term structural and functional trends remain to be evaluated. Because multiple correlation tests were performed without formal multiplicity adjustment, weaker associations may reflect inflation of type I error. In addition, more granular analyses, such as mapping sectoral macular GCL change to localized/pointwise visual field defect patterns and evaluating predictors of long-term functional recovery (e.g., disease duration, opening pressure, medication dose), were not feasible within the available extracted dataset and revision constraints. Finally, this cohort represents a vision-threatening, papilledema-dominant IIH phenotype selected for ONSF, with high baseline Frisén grades and partially preserved central acuity. Findings may not generalize to milder IIH managed medically, and selection bias is inherent given that ONSF is typically reserved for patients at visual risk. Despite these limitations, this study adds to the growing body of evidence supporting the use of OCT in guiding and monitoring ONSF outcomes. The integration of RNFL, macular, and functional data allows for a more comprehensive assessment of therapeutic response.

Future prospective, stratified studies should compare ONSF vs VP shunt vs VSS in complex phenotypes (severe/rapid papilledema, coexisting venous stenosis, obesity), using uniform entry criteria (Modified Dandy), standardized Frisén + OCT RNFL/GCL and HVF endpoints, and reporting visual rescue, headache outcomes, revision/complication rates, and quality of life. Contemporary meta-analyses/guidelines underscore variability and the need for protocolized selection and timing.

## 5 Conclusion

This study demonstrated strengthening structure–function concordance between OCT-derived structural measures and visual field indices over 24 weeks following ONSF in vision-threatening IIH. Early postoperative RNFL measurements may be confounded by edema, whereas later RNFL and macular GCL metrics more closely reflect axonal status and align with visual function. Structural parameters such as RNFL thickness and GCL+ thickness showed increasing correlations with visual field indices over 24 weeks, reflecting ongoing anatomical and functional recovery. Notably, macular volume also correlated with improved retinal sensitivity, underscoring its relevance in visual prognosis. These findings highlight the utility of longitudinal OCT and visual field assessments in tracking recovery and guiding clinical management in IIH patients undergoing ONSF.

## Data Availability

The raw data supporting the conclusions of this article will be made available by the authors, without undue reservation.
